# Novel strain-level resolution of Crohn’s disease mucosa-associated microbiota via an ex vivo combination of microbe culture and metagenomic sequencing

**DOI:** 10.1038/s41396-021-00991-1

**Published:** 2021-05-25

**Authors:** J. J. Teh, E. M. Berendsen, E. C. Hoedt, S. Kang, J. Zhang, F. Zhang, Q. Liu, A. L. Hamilton, A. Wilson-O’Brien, J. Ching, J. J. Y. Sung, J. Yu, S. C. Ng, M. A. Kamm, M. Morrison

**Affiliations:** 1 The University of Queensland Diamantina Institute, Faculty of Medicine, University of Queensland, Woolloongabba, QLD Australia; 2 Department of Medicine and Therapeutics, Institute of Digestive Disease, State Key Laboratory of Digestive Diseases, Li Ka Shing Institute of Health Science, The Chinese University of Hong Kong, Hong Kong, China; 3 Department of Gastroenterology, St Vincent’s Hospital, Melbourne, VIC Australia; 4 Department of Medicine, The University of Melbourne, Melbourne, VIC Australia; 5 Center for Gut Microbiota Research, Department of Medicine and Therapeutics, The Chinese University of Hong Kong, Hong Kong, China; 6 Present Address: Wacker Biotech B.V., Amsterdam, The Netherlands; 7 Present Address: NHMRC Centre of Research Excellence (CRE) in Digestive Health, Hunter Medical Research Institute (HMRI), Newcastle, NSW Australia; 8 Present Address: Lee Kong Chian School of Medicine, Nanyang Technological University, Singapore, Singapore

**Keywords:** Biomarkers, Microbiome, Next-generation sequencing, Clinical microbiology, Metagenomics

## Abstract

The mucosa-associated microbiota is widely recognized as a potential trigger for Crohn’s disease pathophysiology but remains largely uncharacterised beyond its taxonomic composition. Unlike stool microbiota, the functional characterisation of these communities using current DNA/RNA sequencing approaches remains constrained by the relatively small microbial density on tissue, and the overwhelming amount of human DNA recovered during sample preparation. Here, we have used a novel ex vivo approach that combines microbe culture from anaerobically preserved tissue with metagenome sequencing (MC-MGS) to reveal patient-specific and strain-level differences among these communities in post-operative Crohn’s disease patients. The 16 S rRNA gene amplicon profiles showed these cultures provide a representative and holistic representation of the mucosa-associated microbiota, and MC-MGS produced both high quality metagenome-assembled genomes of recovered novel bacterial lineages. The MC-MGS approach also produced a strain-level resolution of key *Enterobacteriacea* and their associated virulence factors and revealed that urease activity underpins a key and diverse metabolic guild in these communities, which was confirmed by culture-based studies with axenic cultures. Collectively, these findings using MC-MGS show that the Crohn’s disease mucosa-associated microbiota possesses taxonomic and functional attributes that are highly individualistic, borne at least in part by novel bacterial lineages not readily isolated or characterised from stool samples using current sequencing approaches.

## Introduction

A step advance in human microbiome research was catalysed by 16 S rRNA gene amplicon sequencing. For the last two decades, this approach has produced a characterisation of the taxonomic profiles of stool and more recently, the mucosa-associated microbiota (MAM) in health and disease [1]. This is exemplified by the large number of case-control and observational studies of the stool and MAM from new-onset and chronic Crohn’s disease (CD) patients, which invariably show a decrease in bacterial diversity coincident with disease and in comparison to non-CD ‘healthy’ subjects. In contrast, longitudinal studies of disease course and the gut microbiome of CD patients are relatively uncommon. In that context, the Post-Operative Crohn’s Endoscopic Recurrence (POCER) study [2] incorporated a longitudinal taxonomic assessment of the MAM from the time of surgery and post-operatively, which identified both ‘protective’ (*Faecalibacterium prausnitzii*) and ‘pathogenic’ (*Proteus* spp.) bacteria in CD remission and recurrence, respectively [3, 4]. Although recent ‘multi-omics’ approaches have provided further insights beyond 16 S rRNA gene-based profiling [5, 6], much still needs to be learned about the microbial biology and functional implications of the MAM and in particular, its involvement with respect to disease location, severity, therapeutic modality (e.g., exclusive enteral nutrition or biologic drugs) and patient immune status [7].

In recent years, metagenomic sequencing (MGS) has greatly empowered gut microbiome studies, by enabling the identification of key microbial functional attributes that are associated with health and disease, including the reconstruction of microbial genomes from these datasets, and species/strain level of resolution of community members (e.g., [8, 9]). However, while MGS has emerged as the preferred approach with samples that are rich in microbial biomass (e.g., stool), constraints remain to the effective use of MGS with DNA from samples with limited microbial density and/or rich in non-microbial (host) DNA. For instance, the total DNA extracted from gut mucosal biopsy samples is often ≥99% host-derived [10], and methods designed to subtractively enrich microbial DNA from these types of samples have limited efficacy and/or still require very large sequencing depths, which is cost prohibitive for many studies [11–15].

Here, we use an ex vivo combination of microbe culture from cryopreserved tissue with shotgun metagenomic sequencing (MC-MGS) to provide a more holistic and functional characterisation of the MAM in post-operative Crohn’s disease patients. The biological veracity of the method was first established via the comparison of the 16 S rRNA gene amplicon profiles produced from DNA extracted directly from biopsy tissues, with those produced from the microbial cultures produced from patient-matched tissue samples. Next, we used these same DNA to produce MGS datasets and, despite some improvement in recovery of microbial data following the subtraction of human DNA, the MC-MGS datasets were substantially more comprehensive, revealing that urea metabolism represents a key and diverse metabolic guild in CD-MAM, and the identification of ‘novel’ lineages of bacteria inherent to these communities.

## Materials and methods

### Samples

Two adjacent pinch biopsy samples were collected from five post-operative CD patients at three sites: at (ANA) and below (BA) the anastomosis site, and from the rectum (REC). One biopsy from each site was immediately transferred into a screw-capped cryotube containing RNAlater. The second biopsy was transferred into a 7 ml glass serum bottle containing 3 ml of an anaerobically prepared, sterilized cryopreservative solution containing (per 100 ml): 0.023 g K_2_HPO_4_, 0.023 g KH_2_PO_4_, 0.023 g (NH_4_)_2_SO_4_, 0.046 g NaCl, 0.01 g MgSO_4_.7H_2_O, 0.007 g CaCl_2_, 0.1 g NaHCO_3_, 0.1 g L-cysteine HCl, 0.1 mg resazurin and 30% (v/v) glycerol. The tissue samples within their respective preservative solutions were stored at -80 °C until later use.

### Microbial culture from biopsy tissue

Modified M2 medium [16] was used for microbial cultivation, which contained (per 100 ml): 1.0 g NZ-Amine, 0.25 g yeast extract, 0.1 g L-cysteine HCl, 0.1 mg resazurin, 0.045 g K_2_HPO_4_, 0.045 g KH_2_PO_4_, 0.045 g (NH_4_)_2_SO_4_, 0.09 g NaCl, 0.01 g MgSO_4_.7H_2_O, 0.012 g CaCl_2_ and 10 ml of clarified rumen fluid. In addition, the medium was supplemented with either a ‘diet-based’ carbohydrate mixture (M2Diet) consisting of D-glucose, soluble starch and D-(+)-cellobiose (0.2% w/v each) or ‘host-derived’ carbohydrates (M2Host) consisting of mucin (porcine stomach), L-fucose, N-acetyl-D-galactosamine, N-acetyl-D-glucosamine (0.2% w/v each) and N-acetylneuraminic acid (0.1% w/v). All carbohydrates were sourced as powders from Sigma Aldrich (MO, USA). For primary microbial cultures using matched biopsies collected from each patient at the site of anastomosis, or the rectum, the cryopreserved tissue was aseptically inoculated into Hungate tubes containing 10 ml of M2Diet medium, with an anaerobic atmosphere (N_2_:CO_2_:H_2_, 85:10:5) and maintained at 37^o^C in a water bath for 24 h. These primary cultures were preserved by adding 3 ml aliquots to 3 ml of the cryopreservative solution as described above, and stored at −80^o^C. In a later series of experiments, a biopsy collected from each patient below the site of anastomosis was transferred to 10 ml M2Host medium in Hungate tubes, also prepared with an anaerobic atmosphere (N_2_:CO_2_:H_2_, 85:10:5) and maintained at 37^o^C in a water bath for 24 hours. At the end of 24 h, 0.1 ml of these cultures were transferred into 10 ml of the M2Diet medium, to generate a secondary microbial-culture and 3 ml added to an equal volume of the cryopreservative solution and stored at −80^o^C. Concurrently, 0.1 ml of the cryopreserved M2Diet cultures described above were used to provide secondary cultures using the M2Host medium. At the end of each culture step, 0.5 ml aliquots were recovered, centrifuged and stored at −30^o^C before DNA extraction.

### DNA extraction and sequencing approaches

DNA was extracted from biopsy tissues and microbial biomass using the repeated bead beating protocol combined with an automated column-based purification system (Maxwell®16) [17] and quantified using the Promega QuantiFluor® dsDNA System (Promega, WI, USA). A 200 ng subsample of the biopsy DNA was then processed with the NEBNext® Microbiome DNA Enrichment Kit (New England Biolabs, MA, USA) according to manufacturers’ instructions. The extracted DNA samples were then sent to the University of Queensland’s Australian Centre for Ecogenomics (www.ecogenomic.org). Here, dual-indexed 16 S rRNA gene amplicon libraries spanning the V4 hypervariable region were constructed with the iTag_515F (GTGYCAGCMGCCGCGGTAA) and iTag_806R (GACTACHVGGGTATCTAATCC) primers. PCR products of ~466 bp were amplified according to the specified workflow with an alteration in polymerase used to substitute NEBNext^®^ Ultra™ II Q5^®^ Mastermix (New England Biolabs #M0544) in standard PCR conditions for 25 cycles. Resulting PCR amplicons were purified using Agencourt AMPure XP beads (Beckman Coulter). Purified DNA was indexed with unique 8 bp barcodes using the Illumina Nextera XT 384 sample Index Kit A-D (Illumina FC-131–1002) in standard PCR conditions for 8 cycles with NEBNext® Ultra™ II Q5® Mastermix. Indexed amplicons were pooled together in equimolar concentrations and sequenced on MiSeq Sequencing System (Illumina) using paired end sequencing with V3 300 bp chemistry in the Australian Centre for Ecogenomics according to manufacturer’s protocol. Positive controls were from a known mock community to monitor for bias in the amplicon library construction, while negative amplification controls were from a mock processed reagent control to monitor for contamination in DNA extraction and library construction. For shotgun metagenomic sequencing (MGS), libraries were constructed using the Illumina Nextera XT DNA Library Preparation Kit (CA, USA), and with MGS sequencing performed with the Illumina NextSeq500 platform as 150 bp paired-end reads, to a sequencing depth of 3 Gbp per sample. Protocols for library preparation, sequencing and data quality assessments follow protocols developed and used by the Australian Centre for Ecogenomics (www.ecogenomic.org).

### 16 S rRNA gene amplicon sequencing data processing and analyses

The 16 S rRNA gene amplicon sequences were processed using the Quantitative Insights into Microbial Ecology (QIIME) pipeline (version 1.9.1) [18], where forward and reverse sequence reads were joined, demultiplexed and quality filtered using a Phred quality score cut-off of ≥20. The Greengenes database (version 13.8) [19] was then used to remove chimeric sequences through USEARCH (version 6.1.544) [20] and subsequently for assigning Operational Taxonomic Units (OTUs) via open reference picking at 97% sequence identity. The resultant OTU table was then filtered to exclude OTUs below 0.1% relative abundance, and OTUs present in the negative controls. Four samples (CD10_REC_Biopsy, CD10_REC_Enriched, CD14_REC_Enriched and CD33_REC_Enriched) produced <1000 reads each, and were excluded from the subsequent analyses. The filtered OTU table was then normalized using cumulative sum scaling (CSS). The normalized OTU table was used to generate Bray-Curtis, weighted and unweighted UniFrac Principal Coordinates Analysis (PCoA) plots and Sparse Partial Least Squares Discriminant Analysis (sPLS-DA) plots using the Calypso (version 8.72) [21] visualization tool and presence/absence plots were generated using the pheatmaps package (https://CRAN.R-project.org/package=pheatmap) available on the R Studio platform (www.rstudio.com).

### Assessment of bacterial DNA enrichment from biopsy DNA

To measure the efficacy of the microbial enrichment step, three independent methods were used. First, we used a qPCR-based quantification of the bacterial 16 S rRNA gene, relative to the copy number of the human β-actin gene present in biopsy DNA before and after microbial enrichment, as described by Shanahan *et al*. [22]. The qPCR was performed using the QuantStudio™ 7 Flex Real-Time PCR System platform (Applied Biosystems, CA, USA), with the Power SYBR Green Master Mix (Life Technologies, UK). Next, we used two different bioinformatics approaches to quantify enrichment. An initial assessment of the raw, unfiltered sequences was performed using GraftM [23] to provide an absolute count on the number of reads assigned to bacterial/archaeal 16 S rRNA genes. Subsequently, we also used MetaPhlAn2 [24] with the quality-filtered MGS data (as described below) to again provide an absolute count of those reads assigned to specific microbial marker genes present in the different MGS datasets.

### MGS data processing

Paired-end raw shotgun sequences generated from ACE were put through Trimmomatic (version 0.36) [25] to remove low quality reads and adaptor sequences. An average of 24 million quality filtered paired-end reads per sample were produced, ranging between 12.7 and 47.4 million reads across all samples, while 2 samples (CD14_REC_Enriched and CD33_REC_Enriched) were excluded due to poor sequencing depth. The quality-filtered paired-end reads were concatenated into a single file and then subjected to analyses via the HUMAaN2 pipeline [26], with taxonomic identification and classification using the MetaPhlAn2 analysis module [24], and with functional characterisation via ChocoPhlAn as the reference pangenome database and UniRef90 [27] as the protein reference database. The identified KEGG Orthologs were mapped to BRITE Hierarchy [28] using custom R scripts, while the between-class analysis (BCA) based on BRITE Hierarchy profiles and Monte Carlo permutation tests was performed using the ‘Ade4’ package available on R. The representative taxonomy and functional plots and heatmaps were generated using the ‘pheatmaps’ package available on R.

### Construction of Metagenome-assembled genomes (MAGs)

Quality-filtered paired-end shotgun sequence reads from the MC-MGS datasets (*n* = 6 per patient) were used to create patient-specific metagenome assemblies using MEGAHIT (version 1.1.1) [29]. Contiguous sequences (contigs) were assembled using BamM (version 1.7.3, available at: http://ecogenomics.github.io/BamM/) and metagenomic binning was performed using MetaBAT (version 0.32.4) [30]. Estimation of the completeness and contamination of the MAGs was performed using CheckM (version 1.0.7) [31]. High-quality MAGs (≥90% completeness and ≤5% contamination scores) and mid-quality MAGs (50–89% completeness and ≤ 10% contamination scores) were then uploaded to Pathosystems Resource Integration Center (PATRIC) [32] to determine whole-genome nucleotide similarity estimation using MinHash (Mash) [33] based on hierarchical clustering to public genomes available on PATRIC (Mash distance < 0.05). Gene annotations were performed using Prokka [34].

### Comparative genomics analyses of MAGs

The Genome Taxonomy Database Toolkit (GTDB-Tk) (version 1.1.1) [35] was used to predict whole genome phylogeny and taxonomic classification of the 47 high-quality MAGs based on the concatenation of 120 bacterial marker genes. The resultant output was then used to build a phylogenetic tree using FastTree (version 2.1.10) [36]. The JSpeciesWS webserver [37] was used for the pairwise genome comparison of Average Nucleotide Identity (ANI) percentages between the *Escherichia coli* MAGs. The *E. coli* MAGs were then functionally annotated based on KEGG Orthology [28] and were subsequently split into CD remission (Rutgeerts score i0/i1) or CD recurrence (Rutgeerts score i2) groups to determine differential KOs via the EnrichM comparative genomics pipeline (version 0.4.9) [38].

### Isolation of urease-positive bacteria

Glycerol stocks of M2Diet cultures from the anastomotic biopsies of patients CD10, CD33 and CD34 were subjected to a ten-fold serial dilution and plated onto MacConkey (Oxoid Ltd, UK) and/or CLED (Cysteine-, Lactose-, Electrolyte deficient) agar (Oxoid Ltd, UK) under aerobic conditions. Single colonies were picked and replated to test for urease-positivity with Urea Agar Base plates with added Phenol Red; and positivity was confirmed by the coordinate pH shift and colour change surrounding these individual colonies. Positive candidate urease-producing colonies were replated onto habitat-simulating M2Diet agar to produce axenic cultures and taxonomically identified using Sanger sequencing based on the 16 S rRNA gene.

## Results

In this study, anastomotic and rectal biopsies from five CD patients were collected 6 months post-surgery during colonoscopy as part of the POCER study [3] and transferred to either RNAlater (for DNA extraction), or a sterile, anaerobically prepared glycerol buffer as a cryopreservative for MC-MGS and stored at −80^o^C. A subsample of the DNA extracted directly from biopsy tissue samples was also subjected to microbial DNA enrichment. This additional step resulted in a twofold to eightfold increase in microbial DNA relative to total biopsy DNA in four out of the five patient samples, as measured by the qPCR-based quantification and two independent bioinformatics approaches as outlined in the Materials and Methods section (Table 1). However, the impact of the DNA enrichment step on the capture of the functional context of the MAM was small (Supplementary Table 2). The mean number of gene families identified by UniRef90 increased from 18,959 to 23,315 for the MGS datasets produced from biopsy DNA pre- and post-enrichment, respectively. In contrast though, the MC-MGS datasets produced on average 78,704 gene families. Interestingly, while the MC-MGS data did not contain human DNA sequences, approximately one-third of the dataset remained unassigned after both taxonomic and functional assignment. These findings suggest our microbial cultures are comprised not only of strains closely related to the reference genomes used in the HUMAaN2 pipeline, but a relatively large proportion of strains possessing novel microbial functionalities and gene diversity.

**Table 1:** The enrichment of microbial reads recovered from anastomotic biopsy DNA samples following the subtractive removal of human DNA, measured by either the fold-change in the fractional ratio of 16 S:β-actin genes assessed by qPCR or by the fold-change in absolute counts of 16 S rRNA gene amplicon reads in unfiltered MGS data using GraftM or quality-filtered MGS data using MetaPhlAn2.

Patient ID	Fold-increase in microbial reads following NEBNext treatment		
	qPCR	GraftM	MetaPhlAn2
CD10	2.2	5.7	7.3
CD12	8.4	5.9	8.8
CD14	2.6	0.9	0.2
CD33	5.2	6.8	6.9
CD34	5.2	5.7	5.2

**Fig. 1:**
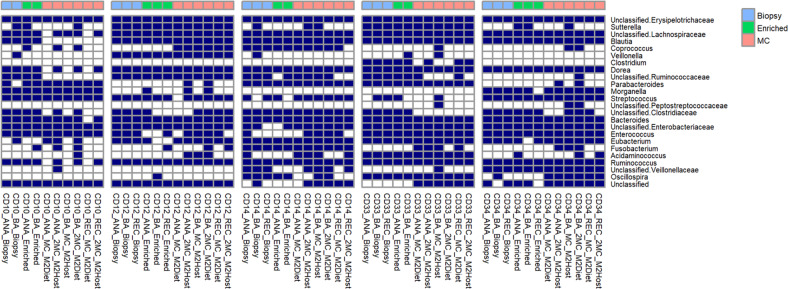
The mucosa-associated microbiota profiles produced from microbial cultures are very similar to those produced using culture-independent methods. The 16 S rRNA gene amplicon profiles were generated using either DNA extracted directly from biopsy tissue (Biopsy), the same DNA subjected to subtractive enrichment of microbial DNA (Enriched), or microbial cultures produced from matched biopsy samples (MC) using either a mixture of diet-based (M2Diet) or host-based (M2Host) carbohydrates (see Methods for more details). Biopsies were collected from each patient at the anastomotic (ANA) or below the anastomotic site (BA), as well as the rectum (REC). The shaded boxes denote those bacterial taxa present in the datasets at >0.1% relative abundance.

**Fig. 2:**
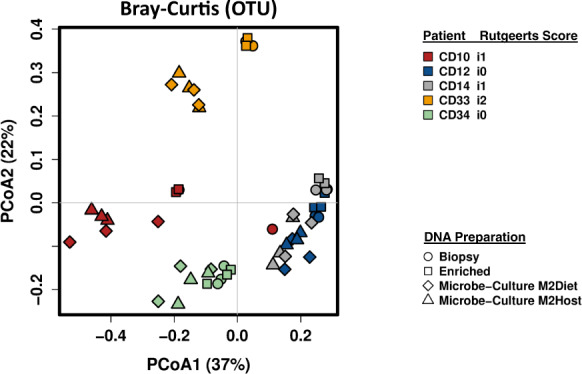
Principal coordinates analysis (PCoA) of mucosa-associated microbiota profiles based on OTU-level Bray–Curtis dissimilarity shows a patient-specific rather than DNA-based clustering of samples. The lack of aggregation of the community profiles arising from the microbial cultures further supports that the patient-specific diversity of the mucosa-associated microbiota is retained in these samples.

All the cultures propagated from the matched biopsy tissue samples produced vigorous growth on both types of media used in this study. After 24 h incubation at 37^o^C, a subsample of these cultures were either stored in the same cryopreservative solution, or microbial biomass was harvested and total DNA extracted and subjected to 16 S rRNA gene amplicon profiling, as described above. The taxonomic profiles produced from these three different DNA samples are shown in Fig. 1, and were comprised of bacterial taxa typically reported in similar studies [1–4]. Furthermore, the community profiles arising from the M2Diet or M2Host cultures showed a high degree of similarity to those produced directly from total and microbe-enriched biopsy DNA. These findings are further illustrated by the PCoA plots based on OTU-level Bray–Curtis dissimilarity (Fig. 2) as well as the unweighted and weighted UniFrac distances (Supplementary Fig. 1), where the patient-based rather than method-based clustering of the MAM profiles generated from three types of DNA preparations are clearly evident. Importantly, the profiles confirmed the cultures possess a diverse collection of anaerobic bacteria including *Lachnospiraceae, Ruminococcaceae, Oscillospira, Unc_Veillonellaceae*, as well as *Clostridium, Bacteroides* and *Fusobacterium*. We also compared the relative abundances of the bacterial taxa present in microbial cultures to those produced from total biopsy DNA. The significant differences are shown in Supplementary Table 3, and show the microbial cultures produced from the 5 subjects do possess small reductions in the relative abundances of some taxa (e.g., *Dorea* spp. from subject CD10 and CD34, and *Blautia* spp. from subjects CD14, CD33 and CD34), whereas others show an increase in their relative abundance (e.g., *Streptococcus* spp. in CD14 and *Enterococcus* spp. and Unclassified*_Enterobacteriaceae* in CD33). However, the changes observed do not suggest there is a broad scale loss, or overgrowth, of bacterial taxa from the initial consortia generated from biopsy tissue. In summation, these results show our approach provides an excellent recovery and retention of the MAM from each CD patient at least upon their initial culture, and thereby, these cultures are amenable to MGS to provide a deep functional understanding of the MAM in these patients.

**Fig. 3:**
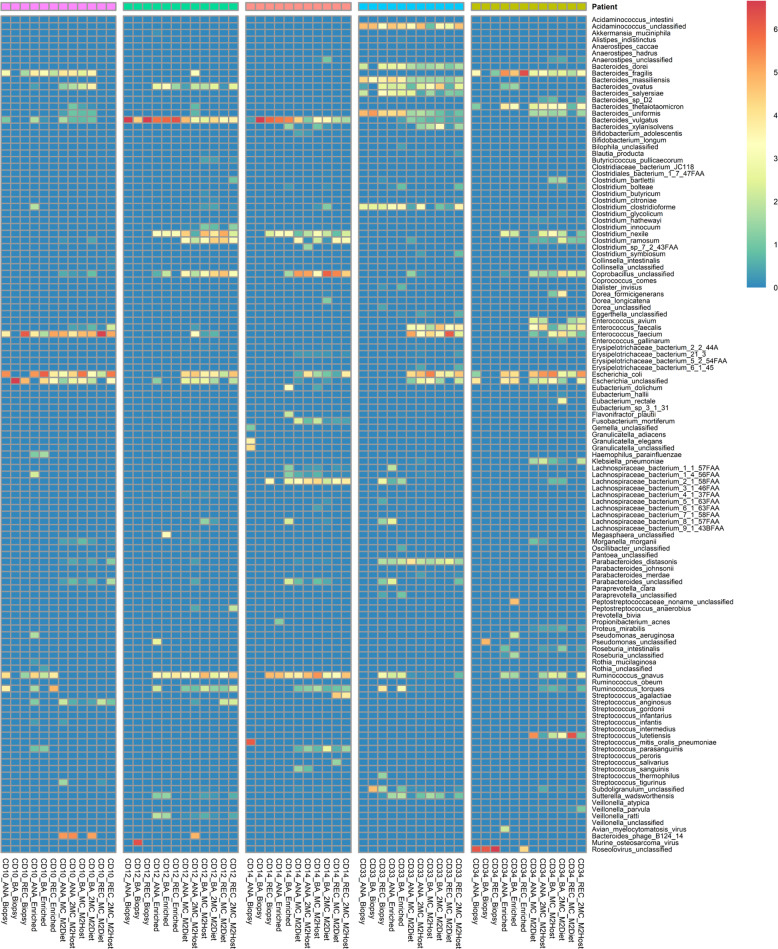
The MC-MGS approach produces a high-resolution footprint of the mucosa-associated microbiota compared to direct sequencing approaches. The taxonomic profiles arising from MetaPhlAn2 analysis of the 3 Gbp MGS datasets produced using either total biopsy DNA (Biopsy), the same DNA subjected to subtractive enrichment of microbial DNA (Enriched), or the microbial cultures produced from matched biopsy samples (MC) using either a mixture of diet-based (M2Diet) or host-based (M2Host) carbohydrates (see Methods for more details). Denoted values are represented as log2-transformed relative abundances.

**Fig. 4:**
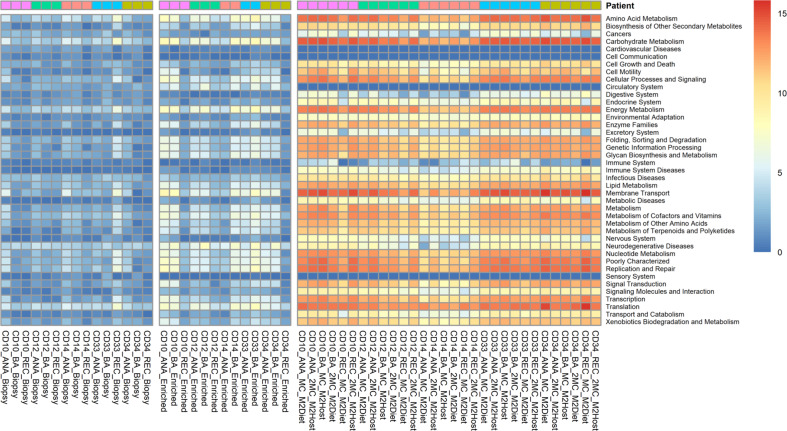
Heatmap of KEGG BRITE Hierarchy levels represented as log2-transformed relative abundance counts per million (cpm), stratified according to DNA-type. Although the microbial enrichment step (Enriched) did increase the amount of functional information from MGS as compared to the Biopsy group, the MC-MGS dataset provided a much more holistic assessment of the CD-MAM functional characteristics.

**Fig. 5:**
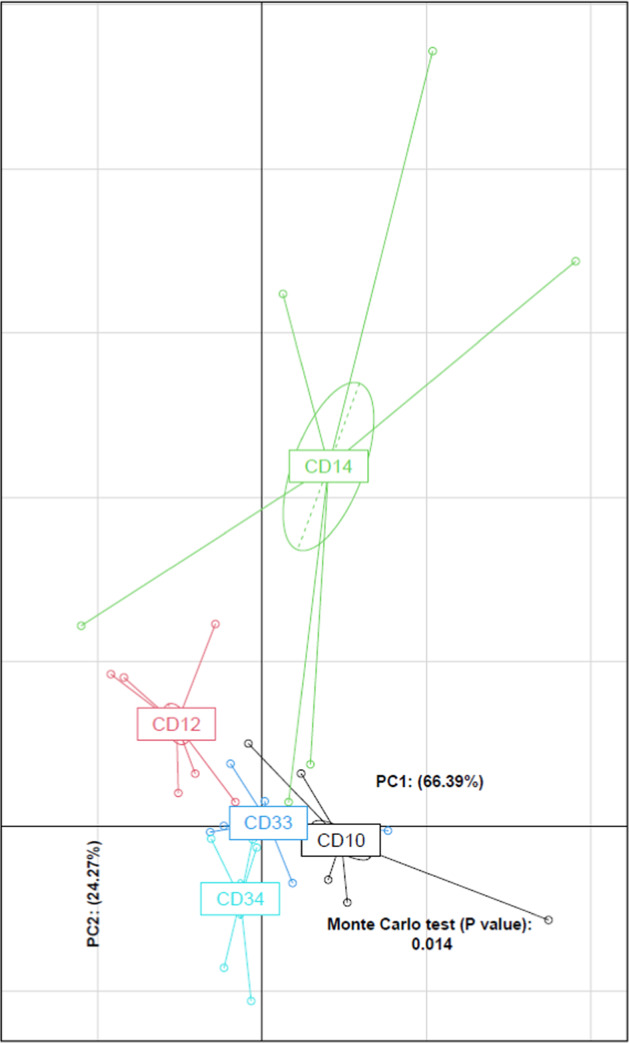
Principle component analysis (PCA) plot generated through between-class analysis (BCA) of identified KEGG BRITE hierarchy functional pathways of the MC-MGS dataset (*n* = 30) and coloured according to patient. The identified functional pathways were shown to retain the patient-specificity as observed in the MGS taxonomic data (Fig. 3).

To that end, all the DNA samples were then sequenced to a depth of 3 Gbp, and the MetaPhlAn2 analysis of these datasets showed that the subtractive enrichment of microbial DNA prior to sequencing did increase the number of microbial reads for 4/5 samples (Table 1). However, the relative abundance of reads produced from host DNA in both types of samples remained very high (~99%, Supplementary Table 2). As such, the comprehensiveness and resolution of the MGS data of microbial origin produced at this sequencing depth remain a major constraint to community characterisation. In that context, and unlike the 16 S rRNA gene amplicon profiles, the MetaPhlAn2 analyses shows the microbial diversity recovered in the MGS data from each type of DNA preparation are dramatically different in terms of their richness (MC-MGS > microbe-enriched MGS > total biopsy MGS, Fig. 3). Only the MC-MGS data retained the inter-subject variations evident from the 16 S rRNA gene amplicon profiles, and this was also evident in the profiles of the HUMAaN2-identified functional pathways at the KEGG BRITE Hierarchy level. The microbial communities recovered from the 5 subjects appear functionally quite similar for many categories (Fig. 4), and although we have not subjected to a thorough statistical analysis due to limited sample numbers, the Principle Component Analysis (PCA) plot generated using Between-Class Analysis (BCA) of the identified KEGG BRITE hierarchy functional pathways (Fig. 5) clearly shows the functional profiles are not only patient-specific but also significantly different to each other (Monte Carlo test, *p* = 0.014).

**Fig. 6:**
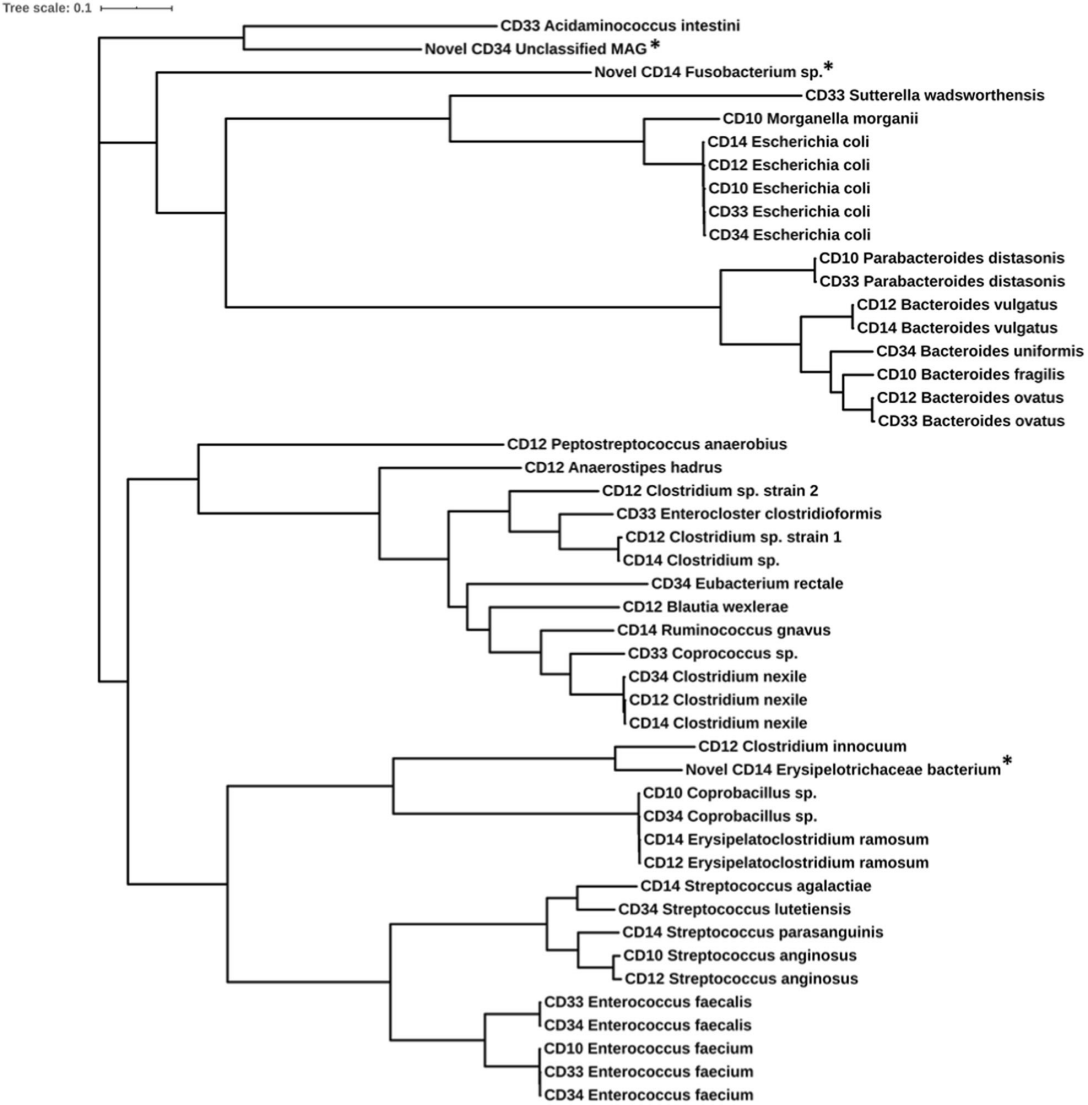
The genome-based phylogenetic tree of all 47 high-quality MAGs constructed based on 120 bacterial marker genes identified using Genome Taxonomy Database Toolkit (GTDB-Tk). Three of the 47 MAGs (denoted by *) were determined to be novel, and were found to cluster separately from the other 44 MAGs.

The 3 Gbp sequencing depth also supported the recovery of 47 unique, high-quality Metagenome Assembled Genomes (MAGs, Table 2) and another 11 mid-quality MAGs (Supplementary Table 4) from the MC-MGS data. The phylogenetic tree of these 47 MAGs generated using GTDB bacterial marker genes revealed 3 MAGs are phylogenetically distinct (Fig. 6). Further analysis using GTDB-Tk showed these 3 MAGs were either unable to be taxonomically identified by FastANI, or have ANI scores of <95% relative to their closest reference genome. One of these MAGs represents a novel lineage within the order *Bacillales*, which by GTDB annotation is proposed to be *Massilibacillaceae* fam. nov. The other two are novel lineages currently affiliated with the *Absiella* and *Fusobacterium* genera (Supplementary Table 5).

**Table 2:** High quality metagenome-assembled genomes (MAGs) recovered from the MC-MGS datasets, grouped according to the individual patient.

CD10	CD12	CD14	CD33	CD34
*Bacteroides fragilis*^a^	*Anaerostipes hadrus*^a^	*Bacteroides vulgatus*	*Acidaminococcus intestini*	*Bacteroides uniformis*^a^
*Coprobacillus* sp.	*Bacteroides ovatus*^a^	*Clostridium nexile*	*Bacteroides ovatus*	*Clostridium nexile*
*Enterococcus faecium*	*Bacteroides vulgatus*	*Erysipelatoclostridium ramosum*	*Enterocloster clostridioformis*	*Coprobacillus* sp.
*Escherichia coli*	***Blautia wexlerae***	*Clostridium* sp.	*Coprococcus* sp.^a^	*Enterococcus faecalis*
***Morganella morganii***	*Clostridium innocuum*	*Escherichia coli*	*Enterococcus faecalis*	*Enterococcus faecium*
*Parabacteroides distasonis*^a^	*Clostridium nexile*	Novel *Erysipelotrichaceae* bacterium	*Enterococcus faecium*	*Escherichia coli*^a^
*Streptococcus anginosus*	*Erysipelatoclostridium ramosum*	**Novel** ***Fusobacterium*** **sp***.*	*Escherichia coli*	*Eubacterium rectale*
	*Clostridium* sp. (1)	*Ruminococcus gnavus*^a^	*Parabacteroides distasonis*^a^	Novel Unclassified MAG
	*Clostridium* sp. (2)	*Streptococcus agalactiae*	*Sutterella wadsworthensis*^a^	*Streptococcus lutetiensis*
	*Escherichia coli*^a^	*Streptococcus parasanguinis*		
	*Peptostreptococcus anaerobius*			
	*Streptococcus anginosus*			

High quality MAGs are considered to be those assemblies predicted by CheckM to possess ≥90% completeness and <5% contamination. The MAGs annotated in bold are confirmed to possess urease genes; those annotated with ^a^possess genes encoding accessory functions involved with urea metabolism. Three MAGs were determined to be of novel lineages using the GTDB-Tk genomic analysis tool (Supplementary Table 5).

We also constructed a reference dataset using the 47 high quality MAGs in Table 2 and mapped the filtered individual reads of the MC-MGS cultures from each subject to this dataset using the default settings in Bowtie2 [39]. The number of reads that show some homology with the MAGs ranged from 69 to 99% (Supplementary Table 6) indicative of the MAGs being truly representative of the microbial diversity present in the patient biopsies. In summary, when considered along with our results using the MGS data for taxonomic (Fig. 3) and functional (Figs. 4 and 5) analyses, both the MC-MGS data and the MAGs show the consortia now in culture represent a diverse range of gut bacteria that are not only consistent with the predictions generated by the 16 S rRNA gene amplicon sequencing, but also include previously uncultured bacteria.

The high-quality *Escherichia coli* MAGs recovered from all 5 CD patient biopsy samples provided the opportunity to more closely examine the genetic heterogeneity represented in this patient group. Importantly, the pairwise ANI comparisons among these MAGs ranged between 96 and 98% suggesting none of the *E. coli* MAGs are clonal in origin and represented strain-based variations between each other (Supplementary Table 7). On that basis, we then used EnrichM in an attempt to compare the *E. coli* MAG recovered from subject CD33 (Rutgeerts score of i2) with the *E. coli* MAGs from the other patients (Rutgeerts scores of i0/i1). Although this grouping is somewhat arbitrary, it is interesting to note this analysis revealed the *E. coli* MAG from subject CD33 differs from the others, in particular by its possession of 2 copies of KEGG ortholog K20444 (encoding *rfbC*, an O-antigen biosynthesis protein) and the *sitABC* manganese/iron transport system (Supplementary Table 8).

In view of these findings, aliquots (0.1 ml) of the glycerol stocks of MC-MGS cultures from the anastomotic biopsies of patients CD10, CD33 and CD34 were subjected to a ten-fold serial dilution and plated onto MacConkey and/or CLED (Cysteine-, Lactose-, Electrolyte deficient) agar under aerobic conditions. Single colonies were picked and replated to test for urease-positivity with Urea Agar Base plates with added Phenol Red; and positivity was confirmed by the coordinate pH shift and colour change surrounding these individual colonies. From these initial efforts, more than 30 urease-positive isolates were recovered from these selective cultures, and by 16 S rRNA gene amplicon sequencing include strains of *Klebsiella pneumoniae, Escherichia fergusonii, Morganella morganii* and *Enterococcus faecium*. Isolates confirmed to be *Proteus mirabilis* were also produced from the single patient (CD34) previously found to possess this bacterium.

## Discussion

As expected, the yields of microbial DNA recovered from mucosal tissue biopsies using the direct tissue-based extraction and enrichment methods, and MC-MGS, are very different. However and importantly, these differences had limited effects on the 16 S rRNA gene amplicon profiles produced from the DNA preparations of matched tissue samples, showing that the patient-specific community structure was at least retained, and even augmented by the MC-MGS approach. In their study of treatment naïve pediatric CD subjects, Gevers et al. [1] reported that the 16 S rRNA gene amplicon profiles from the mucosal biopsies was not greatly different between collection sites from the same individual, which is consistent with our findings here, using either DNA extracted directly from biopsy tissue or via microbial culture using tissue from 3 different sites.

The 16 S rRNA gene amplicon profiles (Fig. 1) also show that the MAM of all patients possess bacterial taxa assigned to unclassified lineages of *Erysipelotrichaceae, Lachnospiraceae, Ruminococcaceae* and *Enterobacteriaceae;* as well as *Bacteroides* and *Ruminococcus* spp. In contrast, the large amounts of human-derived DNA present in both total and microbe-enriched DNA preparations from biopsy tissue (>90%) restricted both the amount and resolution of the MGS data to assess the MAM. With limited exceptions (*Ruminococcus torques* and *Ruminococcus gnavus* for CD10, and some *Lachnospiraceae* for CD33) the MC-MGS approach supported the improved resolution and recovery of viable bacteria representing all these lineages (Fig. 3). Subject CD33 appears to have a specific expansion in *Bacteroides* spp. diversity (e.g., *B. dorei* and *B. massiliensis*) and the MGS data shows all the biopsy samples from this patient contain a strain of *Acidaminococcus intestini*, suggesting that it is a notable member of the MAM, as is *E. clostridioformis*, which have all recently been associated with the IBD-MAM and/or specific IBD phenotypes [40–42]. Given that CD33 is the only subject diagnosed with mild post-operative recurrence at the time of tissue collection, we note these differences as evidence to further investigate the potential role(s) of these bacterial taxa in CD recurrence.

Importantly, the HUMAaN2 data statistics as well as the *de novo* MAG assemblies from the MC-MGS datasets show the CD mucosa-associated microbiota contains heretofore unclassified bacteria, present at varying levels of abundance. For instance, all the MC-MGS datasets identify, albeit at low levels (~0.1%) an unclassified strain of the *Subdoligranulum* lineage, which was recently shown to be reduced in IBD and a key node of the multi-omic IBD functional networks presented by Lloyd-Price et al. [5]. We were also able to recover two (one high-quality and one medium-quality) MAGs from two separate patient samples that were taxonomically affiliated with the family *Erysipelotrichaceae*. The involvement of the *Erysipelotrichaceae* in gastrointestinal health and disease remains contradictory [43], which largely arises from the dearth of cultured isolates providing greater resolution of this expansive bacterial family. For instance, taxa assigned to the *Erysipelotrichaceae* were found to be increased in the intestinal lumen of colorectal cancer patients versus healthy controls [44], and in fecal samples from a mouse model of Crohn’s disease [45]. Furthermore, *Clostridium innocuum* are affiliated with the *Erysipelotrichacea* and has been recently implicated in the development of ‘creeping fat’ as a complication associated with CD strictures and fibrosis [46]. In contrast, reductions in the relative abundance of *Erysipelotrichaceae* were reported from patients with both new-onset and recurrent CD [1]. Such disparities will remain and may be due to the lack of cultured representatives, leading to the poor taxonomic characterisation of taxa belonging to the *Erysipelotrichaceae* family. This further highlights the capacity of MC-MGS in not only providing a comprehensive assessment of the gut microbiota community, but also produces valuable resources that facilitate their initial enrichment and opportunities for the axenic cultivation of novel bacteria.

With specific reference to the *Enterobacteriaceae*, the MC-MGS approach verified the increased richness of this lineage in the MAM communities of these patients predicted from the 16 S rRNA gene amplicon profiles, and includes *Morganella morganii, Proteus mirabilis*, *E. coli* and *K. pneumoniae*. Recent multi-omics assessment of IBD shows both *E. coli* and *K. pneumoniae* are governors of key functional networks that are increased in IBD [5], the latter of which is a recognized producer of urease activity (e.g., [47]. We have previously reported as part of the POCER study that detectable levels of *Proteus* spp. are associated with an elevated risk of CD recurrence [4] and more recently, demonstrated a key role for *P. mirabilis* in CD inflammation [48]. Notably, *Proteus* spp. are now phylogenetically assigned to the *Morganellaceae* fam. nov. of which *M. morganii* is a type species [49]. A unifying metabolic trait across members of these bacterial taxa is urea metabolism, which has now been identified as a key functional change coincident with CD dysbiosis; and the inoculation of mice with recombinant, urease-positive strains of *E. coli* MP1 can promote gut dysbiosis through a bloom in *Proteobacteria* abundance [50]. Using this metabolic guild as a selective strategy as described in the Methods, we recovered from the biopsy outgrowth cultures a variety of urease-positive *Proteobacteria* using including *Morganella morganii* (subject CD10), *Proteus mirabilis* (subject CD34) and *Klebsiella pneumoniae* (CD34). The Prokka-based annotations of the MAGs further showed that 14/47 possess genes encoding either for urease, and/or genes encoding accessory functions putatively involved with urea metabolism (e.g., urea transporters, hydrogenase-urease nickel incorporation protein[s], and transcriptional activators). Based on these collective findings, the MC-MGS approach resulted in the production of deep sequence data and MAGs, but also axenic bacterial isolates representing key metabolic guilds, and thereby provides a variety of resources to study the role of the MAM in digestive health and disease.

In conclusion, we have shown here that an ex vivo combination of microbial culture with metagenomic sequencing enables a cost- and time-effective strategy to produce a deep, functional understanding of the mucosa-associated microbiota, where tissue samples contain a relatively small microbial load and/or large quantities of non-microbial (host) DNA. These communities are not only an excellent representation of the communities predicted by 16 S rRNA gene amplicon sequencing, but support the identification and detailed characterisation of tissue-specific ‘microbial dark matter’, as well as strain-level resolution of bacterial taxa present in these communities. For these reasons, MC-MGS can advance our multi-omics understanding of microbial dysbiosis beyond ecological principles applied to taxonomy-based datasets, and also bring genomes to life in the form of defined consortia and/or axenic isolates to enable greater clarity of the role of mucosa-associated microbiota in host-response, inflammation and inflammatory bowel disease.

## Supplementary information

Supplementary Materials

## Data Availability

The raw sequence data and MAGs presented here have been deposited in the NCBI database under BioProject ID PRJNA715634.
