# Supplementary material for: Novel strain-level resolution of Crohn’s disease mucosa-associated microbiota via an ex vivo combination of microbe culture and metagenomic sequencing
Source: ISME J. 2021 May 25;15(11):3326–38. doi: 10.1038/s41396-021-00991-1 (PMC8528831; doi:10.1038/s41396-021-00991-1)
Supplement: Supplementary file 1 — Supplementary Materials [file 41396_2021_991_MOESM1_ESM.docx]

**Supplementary Figure 1: Principal coordinates analysis (PCoA) of mucosa-associated microbiota profiles based on OTU-level weighted and unweighted UniFrac distances shows a patient-specific rather than DNA-based clustering of samples**. The lack of aggregation of the community profiles arising from the microbial cultures further supports that the patient-specific diversity of the mucosa-associated microbiota is retained in these samples.


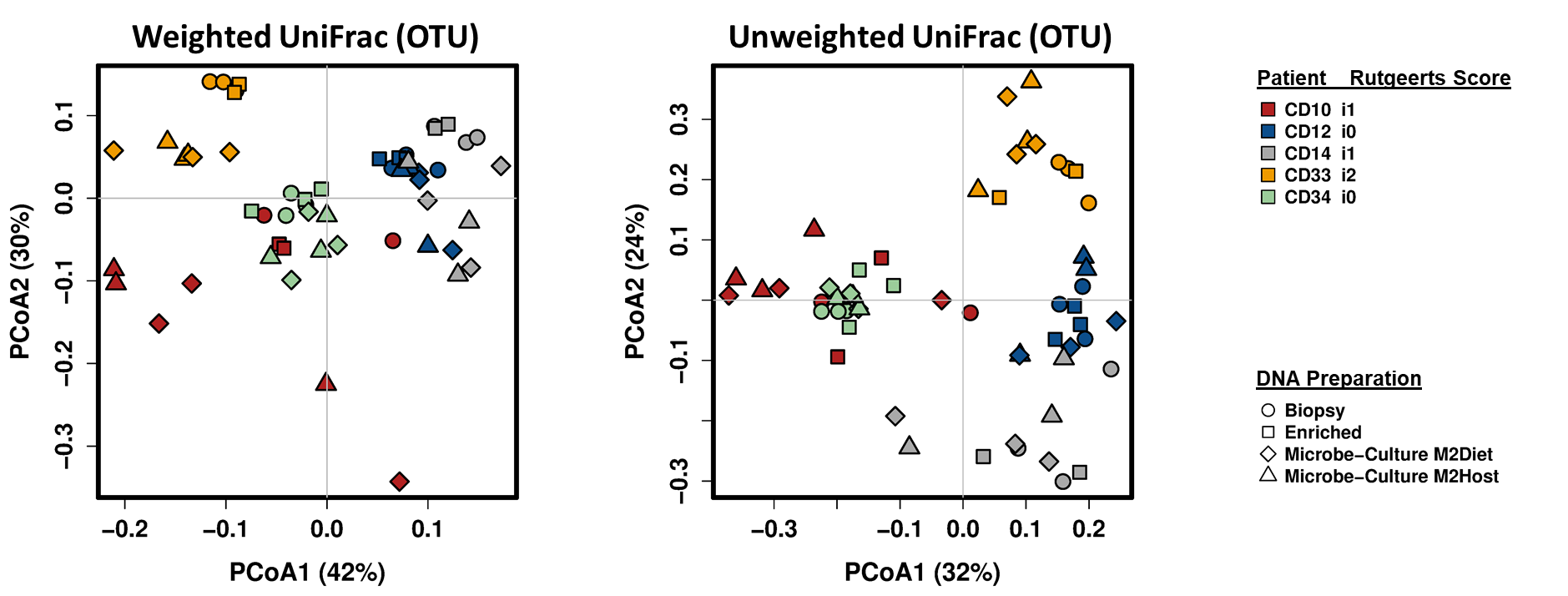


**Supplementary Table 1:** Clinical metadata of the 5 CD patients previously recruited as part of the POCER study.

| **ID** | **Age** | **Gender** | **Indication for Surgery** | **Disease Duration (years)** | **Smoking status** | **Previous Surgery** | **Montreal Classification** | **Initial therapy to 6MO** | **6MO Endoscopy Rutgeerts Score** | **6MO Disease Recurrence** |
| --- | --- | --- | --- | --- | --- | --- | --- | --- | --- | --- |
| CD10 | 76 | Female | Obstruction | 2 | No | No | A3 L1 B2 | Metronidazole (3months) | 1 | No |
| CD12 | 55 | Male | Failure of medical therapy | 2 | No | No | A3 L1 B3 | Adalimumab | 0 | No |
| CD14 | 51 | Female | Perforation | 3 | No | No | A3 L1 B3 | Azathioprine | 1 | No |
| CD33 | 47 | Female | Perforation | 2 | Yes | No | A3 L3 B3 | Azathioprine | 2 | Yes |
| CD34 | 32 | Male | Perforation | 4 | Yes | No | A2 L1 B3 | Azathioprine | 0 | No |

**Supplementary Table 2:** The mean (+/- SEM) number of quality-filtered reads of microbial origin, their relative abundance, number of gene families present in the MGS datasets, as determined using the HUMAnN2 pipeline with UniRef90 as the reference protein database.

| **Sample type** | **Mean (+/- SEM) number**  **of reads assigned to microbial taxa** | **Mean (+/- SEM) abundance (%) of total reads assigned to microbial taxa** | **Mean (+/- SEM) number of gene families** |
| --- | --- | --- | --- |
| Biopsy (n=15) | 26 342 (± 12 370) | 0.10% (± 0.05%) | 19 646 (± 1 834) |
| Enriched (n=13) | 116 822 (± 40 492) | 0.41% (± 0.12%) | 23 315 (± 3 389) |
| MC-MGS (n=30) | 15 061 776 (± 1 204 892) | 65.32% (± 2.74%) | 78 704 (± 4 184) |

**Supplementary Table 3:** Taxa identified to be significantly enriched or repressed (Two-way ANOVA) using the MC-MGS methodology as compared to the community present on the biopsies, based on the 16S-rRNA gene amplicon relative abundances.

| **Patient ID** | **Taxa (Genus level)** | **Bonferroni adjusted p-value** | **Enriched/Suppressed** | **Mean relative abundance in Biopsy group** | **Mean relative abundance in MC group** |
| --- | --- | --- | --- | --- | --- |
| **CD10** | *Dorea* | 0.01 | Suppressed | 12.32% | 1.30% |
|  | Unclassified.*Lachnospiraceae* | 0.04 | Suppressed | 11.52% | 1.61% |
| **CD12** | Unclassified.*Lachnospiraceae* | 0.0002 | Suppressed | 17.11% | 12.12% |
| **CD14** | *Blautia* | 0.01 | Suppressed | 8.34% | 4.29% |
|  | *Streptococcus* | 0.008 | Enriched | 1.61% | 5.77% |
|  | Unclassified.*Lachnospiraceae* | 0.0001 | Suppressed | 13.65% | 6.02% |
|  | Unclassified.*Erysipelotrichaceae* | 0.0001 | Enriched | 3.96% | 9.79% |
| **CD33** | *Blautia* | 0.0006 | Suppressed | 10.22% | 5.60% |
|  | *Enterococcus* | 0.0001 | Enriched | 0.76% | 5.95% |
|  | *Ruminococcus* | 0.001 | Suppressed | 9.78% | 5.33% |
|  | Unclassified.*Clostridiaceae* | 0.007 | Suppressed | 3.93% | 0.00% |
|  | Unclassified.*Enterobacteriaceae* | 0.0002 | Enriched | 2.81% | 7.64% |
|  | Unclassified.*Lachnospiraceae* | 0.0001 | Suppressed | 15.16% | 7.90% |
| **CD34** | *Blautia* | 0.0485 | Suppressed | 9.39% | 6.52% |
|  | *Dorea* | 0.0001 | Suppressed | 21.35% | 13.68% |
|  | Unclassified.*Clostridiaceae* | 0.03 | Suppressed | 4.43% | 1.44% |

**Supplementary Table 4:** Mid-quality metagenome-assembled genomes (MAGs) recovered from the MC-MGS datasets predicted by CheckM to possess between 50% and 90% completeness and <10% contamination, grouped according to individual patient.

| CD10 | CD12 | CD14 | CD33 | | CD34 |
| --- | --- | --- | --- | --- | --- |
| *Bacteroides uniformis* | *Ruminococcus* sp. | *Bacteroides xylanisolvens* | *Bacteroides uniformis* | | *Ruminococcus* sp. |
|  | *Ruminococcus gnavus* | *Dorea longicatena* | *Blautia* sp. | | *Clostridium ramosum* |
|  | *Coprococcus* sp. |  | *Erysipelotrichaceae* bacterium | |  |
|  |  |  | |  |  |

**Supplementary Table 5: High-quality MAGs determined to be novel using the Genome Taxonomy Database Toolkit (GTDB-Tk).** 2 MAGs could only be classified by GTDB-Tk to the genus level, suggesting that they represent potentially novel species, while the other MAG from patient CD34 represents a novel lineage within the *Massilibacillaceae* family level. The FastANI percentages between each MAG to their respective phylogenetically closest neighbor all fall below the species level threshold (< 95%).

| MAG ID | GTDB-Tk classification | FastANI taxonomy | FastANI (%) | Note |
| --- | --- | --- | --- | --- |
| Novel CD14 *Erysipelotrichaceae*bacterium | f__*Erysipelotrichaceae*;g__*Absiella*;s__ | N/A | N/A | taxonomic classification fully defined by topology |
| Novel CD14 *Fusobacterium* sp. | f__*Fusobacteriaceae*;g__*Fusobacterium*_A;s__ | f__*Fusobacteriaceae*;g__*Fusobacterium*_A;s__*Fusobacterium*_A *mortiferum* | 81.91 | taxonomic classification fully defined by topology |
| Novel CD34 Unclassified MAG | f__*Massilibacillaceae*;g__;s__ | N/A | N/A | taxonomic novelty determined using RED |

**Supplementary Table 6: Percentage of reads from the MC-MGS dataset that mapped, using Bowtie2 at default settings, to the reference dataset constructed based on the 47 high-quality MAGs, grouped according to the patient (n=6 for each patient).**

| **Patient ID** | **Percentage of total reads mapped to reference MAGs** | |
| --- | --- | --- |
| CD10 | 82.66% – 91.88% | |
| CD12 | 81.69% – 89.78% | |
| CD14 | 81.66% – 92.67% | |
| CD33 | 78.69% – 89.01% | |
| CD34 | 69.93% – 98.97% | |
|  |  |  |

**Supplementary Table 7: Pairwise comparison of Average Nucleotide Identity (ANI) percentages with MAGs taxonomically identified to be *Escherichia* *coli* from all 5 patients, and a MAG identified to be *Morganella* *morganii* was used as an outgroup.** The whole genome comparison between the 5 *E. coli* MAGs suggests that they are highly genetically similar (> 95% ANI) but there is a small degree of strain-based variation between each other.

|  | | CD10  *Escherichia* *coli* | CD12  *Escherichia* *coli* | CD14  *Escherichia* *coli* | CD33  *Escherichia* *coli* | CD34  *Escherichia* *coli* | CD10  *Morganella* *morganii* |
| --- | --- | --- | --- | --- | --- | --- | --- |
| CD10  *Escherichia* *coli* | | - | 97.15 | 97.14 | 96.97 | 96.45 | 70.52 |
| CD12  *Escherichia* *coli* | | 97.03 | - | 97.56 | 97.84 | 96.59 | 70.29 |
| CD14  *Escherichia* *coli* | | 97.00 | 97.55 | - | 97.36 | 96.46 | 70.25 |
| CD33  *Escherichia* *coli* | | 97.01 | 98.13 | 97.64 | - | 96.53 | 70.33 |
| CD34  *Escherichia* *coli* | | 96.38 | 96.52 | 96.44 | 96.33 | - | 70.42 |
| CD10  *Morganella* *morganii* | | 70.45 | 70.54 | 70.36 | 70.49 | 70.45 | - |
|  |  |  |  |  |  |  |  |

**Supplementary Table 8: EnrichM comparative functional gene analyses of the KEGG Orthologs (KOs) of the 4 *E. coli* MAGs recovered from patients with i0 and i1 Rutgeerts scores as compared to the single *E. coli* MAG recovered from the patient with a Rutgeerts score of i2.**

| **KEGG Orthology** | **Mean count in i0/i1** | | **Count in i2** | **KO description** | |
| --- | --- | --- | --- | --- | --- |
| K01551 | 0 | | 1 | arsA, ASNA1, GET3; arsenite/tail-anchored protein-transporting ATPase [EC:3.6.3.16 3.6.3.-] | |
| K06952 | 0 | | 1 | uncharacterized protein | |
| K07165 | 0 | | 1 | fecR; transmembrane sensor | |
| K10918 | 0 | | 1 | aphB; LysR family transcriptional regulator, transcriptional activator AphB | |
| K11604 | 0 | | 1 | sitA; manganese/iron transport system substrate-binding protein | |
| K11605 | 0 | | 1 | sitB; manganese/iron transport system ATP-binding protein | |
| K11607 | 0 | | 1 | sitC; manganese/iron transport system permease protein | |
| K16091 | 0 | | 1 | fecA; Fe(3+) dicitrate transport protein | |
| K20444 | 0 | | 2 | rfbC; O-antigen biosynthesis protein [EC:2.4.1.-] | |
|  |  |  | | |  |
